# Early Targeting of L-Selectin on Leukocytes Promotes Recovery after Spinal Cord Injury, Implicating Novel Mechanisms of Pathogenesis

**DOI:** 10.1523/ENEURO.0101-18.2018

**Published:** 2018-08-29

**Authors:** D. A. McCreedy, S. Lee, C. J. Sontag, P. Weinstein, A. D. Olivas, A. F. Martinez, T. M. Fandel, A. Trivedi, C. A. Lowell, S. D. Rosen, L. J. Noble-Haeusslein

**Affiliations:** 1 Department of Neurological Surgery, University of California, San Francisco, CA 94143; 2 Brain and Spinal Injury Center, University of California, San Francisco, CA 94110; 3 J. David Gladstone Institutes, University of California, San Francisco, CA 94158; 4 Department of Laboratory Medicine, University of California, San Francisco, CA 94143; 5 Department of Anatomy and Program in Immunology, University of California, San Francisco, CA 94143; 6 Department of Physical Therapy and Rehabilitation Science, University of California, San Francisco, CA 94143

**Keywords:** diclofenac, L-selectin, leukocytes, myelin, oxidative stress, spinal cord injury

## Abstract

L-selectin, a lectin-like receptor on all leukocyte classes, functions in adhesive and signaling roles in the recruitment of myeloid cells from the blood to sites of inflammation. Here, we consider L-selectin as a determinant of neurological recovery in a murine model of spinal cord injury (SCI). Spinal cord-injured, L-selectin knock-out (KO) mice (male) showed improved long-term recovery with greater white matter sparing relative to wild-type (WT) mice and reduced oxidative stress in the injured cord at 72 h post-SCI. There was a partial and transient reduction in accumulation of neutrophils in the injured spinal cords of KOs at 24 h post-injury. To complement these findings with KO mice, we sought a pharmacologic means for lowering L-selectin levels. We found that diclofenac, a nonsteroidal anti-inflammatory drug (NSAID), induced the shedding of L-selectin from the cell surface of myeloid subsets, specifically neutrophils and non-classical monocytes, in the blood and the injured spinal cord. Diclofenac administration to injured WT mice enhanced neurological recovery to a level comparable to that of KOs but did not improve recovery in KOs. While diclofenac treatment had no effect on myeloid cell accumulation, there was a reduction in oxidative stress at 72 h post-SCI. These findings implicate L-selectin in secondary pathogenesis beyond a role in leukocyte recruitment and raise the possibility of repurposing diclofenac for the treatment of SCI.

## Visual Abstract

**Figure:**
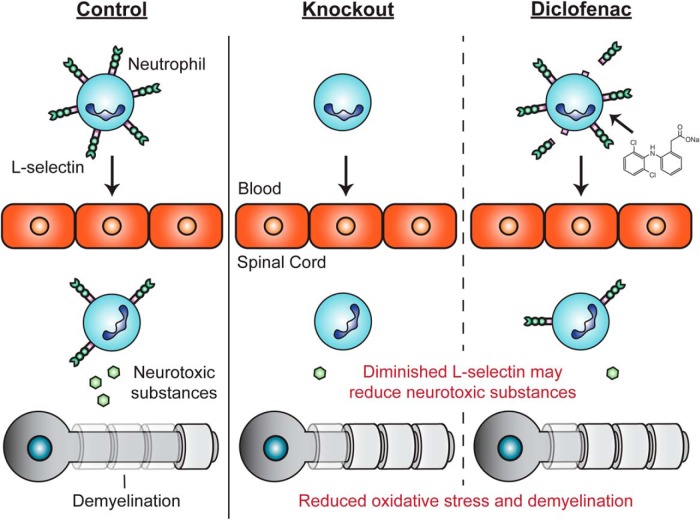

## Significance Statement

In this study, we establish L-selectin, an adhesion and signaling receptor on immune cells, as a determinant of long-term recovery and tissue sparing after spinal cord injury (SCI). We demonstrate that L-selectin contributes to secondary pathogenesis during acute inflammation, and implicate L-selectin in novel roles other than recruitment. We also report a strategy to improve recovery by employing diclofenac, an FDA-approved nonsteroidal anti-inflammatory drug (NSAID) that induces the shedding of L-selectin from the surface of innate immune cells. Our findings demonstrate a critical time-period for anti-inflammatory intervention in a murine model of SCI and suggest that diclofenac be tested as an acute therapy for attenuating neurologic deficits following SCI in humans.

## Introduction

Spinal cord injury (SCI) results in partial or complete loss of motor and sensory function below the site of injury. The neurologic deficits resulting from SCI are not solely attributed to the initial mechanical damage, and there is a broad consensus that early infiltrating leukocytes, primarily myeloid lineage cells (i.e., neutrophils and monocytes), release neurotoxic substances including reactive oxygen species (ROS), proteases, and proinflammatory cytokines that cause secondary tissue damage (Chatzipanteli et al., 2002; Noble et al., 2002; Bareyre and Schwab, 2003; David and Kroner, 2011). Various strategies, directed at attenuating the early recruitment of myeloid cells from the blood into the injured spinal cord, have shown promising results for reducing cell injury and improving long-term neurologic outcomes (Popovich et al., 1999; Gris et al., 2004; Popovich and Longbrake, 2008; Lee et al., 2011; Zhang et al., 2011). However, inconsistent benefits or deleterious consequences have been observed in other studies (Stirling et al., 2009; Hurtado et al., 2012). These discrepancies may be due to opposed reparative and damaging activities in the targeted myeloid subsets. In the present study, we have identified L-selectin, a leukocyte adhesion/signaling receptor, as a novel therapeutic target in SCI.

Selectins are C-type lectins that generally function sequentially with integrins during the multistep process of leukocyte recruitment from the blood into sites of inflammation (Ley et al., 2007; McEver, 2015). The vascular selectins, E- and P-selectin, are upregulated on inflamed vascular endothelium and bind to ligands such as P-selectin glycoprotein ligand-1 (PSGL-1) on leukocytes (McEver, 2015). L-selectin (CD62L) on lymphocytes mediates their rolling on high endothelial venules during homing to secondary lymphoid organs through the interaction of its lectin domain with carbohydrate-based ligands on this specialized endothelium (Rosen, 2004). L-selectin is also broadly expressed on circulating myeloid leukocytes and has adhesive and signaling activities that underlie various responses of these cells to inflammation (Ley et al., 2007; McEver, 2015). Studies employing knock-out (KO) mice or blocking antibodies have demonstrated that L-selectin, working in concert with vascular selectins, is involved in the recruitment of myeloid cells from the blood into various sites of inflammation (Lewinsohn et al., 1987; Pizcueta and Luscinskas, 1994; Tedder et al., 1995; Ley et al., 2007; Zuchtriegel et al., 2015).

It is now clear that L-selectin contributes to myeloid cell recruitment as a secondary adhesion molecule that mediates tethering between endothelial-adherent leukocytes and circulating leukocytes, via binding in trans to PSGL-1 on leukocytes (Walcheck et al., 1996; Sperandio et al., 2003), and as a signaling molecule that augments the activation of integrins on rolling leukocytes (Stadtmann et al., 2013; Morikis et al., 2017). Additional activities, apart from the recruitment of leukocytes from the blood, are indicated in that the ligation of L-selectin on neutrophils by soluble carbohydrate ligands, such as carcinoma-derived or salivary mucins, potentiates the degranulation of these cells (Shao et al., 2011; Mohanty et al., 2015). In the context of CNS inflammation, the possibility of a post-recruitment role for L-selectin has emerged based on the observation that L-selectin mediates the *in vitro* adhesion of leukocytes to myelinated fiber tracts in the CNS (Huang et al., 1991, 1994). In light of its potential recruitment and post-recruitment activities, we have investigated whether the reduction of L-selectin function through genetic or pharmacologic means has an impact on neurologic recovery in a murine model of SCI.

We demonstrate that complete L-selectin deficiency results in a partial reduction of neutrophil accumulation and oxidative stress in the acutely injured cord as well as improved long-term neurologic recovery that corresponds to greater sparing of white matter. We further show that diclofenac, an nonsteroidal anti-inflammatory drug (NSAID) and an inducer of L-selectin shedding from the leukocyte cell surface (Díaz-González et al., 1995; Gómez-Gaviro et al., 2002), has beneficial effects in SCI comparable to the genetic elimination of L-selectin. Since diclofenac is currently approved by the FDA (Altman et al., 2015), there could be an opportunity to repurpose this drug for the spinal cord-injured patient. The beneficial consequences of reducing L-selectin levels cannot be attributed solely to reduced leukocyte recruitment, particularly in the case of diclofenac, highlighting the consideration of L-selectin in novel roles in secondary pathogenesis and subsequent long-term neurologic deficits.

## Materials and Methods

### Animals

These studies were approved by the Institutional Animal Care and Use Committee at the University of California San Francisco and were in accordance with the United States Department of Agriculture guidelines. Homozygous L-selectin KO mice and their wild-type (WT) littermates were generated by breeding heterozygous males and females on a C57Bl/6 background. We confirmed that mice from L-selectin KO and WT colonies did not contain the recently reported copy number variant in the *Dock2* allele (Mahajan et al., 2016). WT and KO littermates were then studied with the exception of flow cytometry experiments where WTs were purchased from The Jackson Laboratory. WT mice for diclofenac studies were purchased from Jackson Laboratories. Mice were housed in groups of two to five before injury and singly housed after SCI.

### SCI

Adult male mice (approximately three to five months of age) were anesthetized with 2.5% Avertin (0.02 ml/g body weight, i.p., tribromoethanol; Sigma) or 2% isoflurane and subjected to a spinal cord contusion injury as described previously (Lee et al., 2011). Briefly, a laminectomy was performed at the ninth thoracic vertebra and a 3-g weight was dropped 5–7.5 cm onto the exposed dura mater to produce the SCI. After injury, the skin was closed with wound clips. Body temperature was maintained at 37°C with a warming blanket throughout the surgery and during recovery from anesthesia. Postoperative care included subcutaneous administration of saline and antibiotics daily for 10 d and manual expression of the bladder twice per day until euthanasia.

### Treatment with diclofenac

Diclofenac (Sigma) was dissolved in PBS at 2.5 mg/ml and sterile filtered before use. To determine whether diclofenac modulates neurologic recovery after SCI, diclofenac (20, 30, or 40 mg/kg) was administered intraperitoneally immediately, 3 h, or 8 h after SCI. The dosing was based on previous studies in rodents (Grace et al., 2001). Behavioral tests were performed as described below.

### Assessment of neurologic recovery

Two behavioral tests, Basso Mouse Scale (BMS) and grid walk, were performed in the same mice to evaluate functional improvements after SCI. The nine-point BMS was used to examine locomotor recovery in an open field (53 × 108 × 5.5 cm; Basso et al., 2006). This rating scale takes into account limb movement, stepping, coordination, and trunk stability. Mice were tested at 1, 3, and 7 d and weekly thereafter until euthanasia at five to six weeks post-SCI. For studies examining diclofenac in WTs, mice achieving a BMS score ≥1 at 1 d post-SCI were considered insufficiently injured and were removed from the analysis. For grid walking, a mouse (with a BMS score of four or greater) was positioned on a grid, divided into 0.5-cm squares, and the number of foot faults was recorded over a period of 3 min. A foot fault was evident when a paw fully extended through a space in the grid. The grid walking test was performed over 3 d at approximately five weeks post-SCI with three trials per day.

### Measurement of white matter sparing

Animals were euthanized at 35 or 42 d post-SCI and perfused with 50 ml of PBS followed by 50 ml of 4% paraformaldehyde (pH 7.4). The spinal cords were removed, postfixed overnight, and cyroprotected in 30% sucrose for 4 d. Cords were then embedded and frozen at -80 °C until sectioning; 20-μm transverse sections were made on a cryostat, and serial sections, 500 μm apart, were chosen for staining of white matter using either luxol fast blue (LFB) or eriochrome cyanine. Sections were evaluated by light microscopy and the one with the least spared white matter was selected as the lesion epicenter. For sections stained with LFB, the area of residual white matter was hand-traced using Neurolucida software (Microbrightfield Bioscience) and the percentage of spared white matter relative to the total cross sectional area of the cord at the epicenter was determined (Lee et al., 2011). This epicenter measurement has previously been demonstrated to correlate with injury severity and the degree of functional recovery (Kuhn and Wrathall, 1998). For sections stained with eriochrome cyanine, the area of residual white matter at the epicenter was traced and quantified using the StereoInvestigator (MBF Bioscience) Cavalieri probe.

### Immunoblotting

A 0.5 cm length of cord, centered over the site of impact and representing the epicenter, was homogenized in Glo lysis buffer (Promega) or RIPA buffer (Thermo Fisher). The protein concentration in homogenates was determined by the BCA protein assay kit (Pierce). For detecting malondialdehyde (MDA), we used the OxiSelect Malondialdehyde immunoblot kit (Cell BioLabs, Inc.) according to the manufacturer’s protocol. Briefly, 20 μg of protein was loaded onto 12–15% SDS-PAGE gel and transferred onto a nitrocellulose or PVDF membrane. After blocking the membrane with 1% BSA solution, the membrane was incubated with rabbit anti-MDA antibody overnight. The membrane was washed with Tris-buffered saline including 0.1% triton X-100 and then incubated with anti-rabbit-HRP antibody for 1 h. MDA-positive bands were detected using Pierce SuperSignal West Pico Chemiluminescent substrate (Thermo Fisher Scientific). Bands at ∼65 kDa, including both bands for uninjured (UN) control samples, were measured. For all of the comparisons, β-actin (Sigma, RRID:AB_476744) or GAPDH (Millipore, RRID:AB_2107426) served as a loading control and was used for normalization.

### Cell isolation and treatments

Neutrophils were isolated from peripheral blood of mice by Ficoll-Paque (Ficoll-Paque PREMIUM 1.084, GE Healthcare Bio-Sciences AB) according to manufacturer’s protocol. Briefly, 2 ml of peripheral blood were obtained by cardiac puncture from three mice and mixed with 10 ml of HBSS. After addition of 3 ml of Ficoll-Paque, the sample as subjected to density-gradient centrifugation of 30 min at 400 × *g* at room temperature (RT). Neutrophils were carefully collected from upper layers. To purify the neutrophil-enriched fraction, erythrocytes were lysed with RBC lysis buffer at RT (eBioscience). The purity of the neutrophil fraction was ≥90%.

To investigate whether NSAIDs induce L-selectin shedding, the isolated cells were treated with NSAIDs as previously described (Gómez-Gaviro et al., 2002). Cells were resuspended in PBS and incubated either alone or in the presence of diclofenac (20–500 µg/ml) or meclofenamic acid (MFA; 20–500 µg/ml, Sigma) for 30 min at 37°C. PMA (250 ng/ml) served as a positive control. All NSAIDs were dissolved in PBS. Then cells were analyzed by flow cytometry and the supernatants were analyzed for soluble L-selectin (sL-selectin) by enzyme-linked immunosorbent assay (sL-selectin ELISA section). Data were collected from seven independent experiments for flow cytometry and two independent experiments for ELISA.

### Flow cytometry

For neutrophils and mononuclear cells exposed to NSAIDs, the cells were washed with PBS then incubated with anti-mouse CD16/32 Fc blocking antibody (1:10 dilution; eBioscience, RRID:AB_467133) for 10 min followed by anti-CD62L Ab conjugated with PE (1:10 dilution, Mel-14 clone, eBioscience, RRID:AB_465720) for 30 min at 4°C. After washing in PBS, the cells were analyzed in a FACScan flow cytometer (Becton Dickinson) and FlowJo software (Tree Star Inc.). In all *in vitro* experiments, at least 10^6^ cells were analyzed for flow cytometry. Cell viability was determined by 7-amino-acinomycin D staining (7-AAD; BD Bioscience). Data were collected from seven independent experiments.

For *in vivo* experiments, blood samples were obtained by cardiac puncture using a heparin-primed syringe from uninjured and spinal cord-injured mice. Recruitment of leukocytes from the blood into spinal cord can occur across blood vessels in the meninges or parenchymal tissue (Ransohoff et al., 2003). Injured mice were perfused with 25 ml of ice cold PBS to remove free leukocytes within blood vessels. From each animal, a 5-mm region of spinal cord (with associated meninges) centered over the injury site was removed and stored in ice cold RPMI media. The spinal cord segment was then mechanically dissociated using a plastic tissue pestle. The suspension was filtered through a 100-µm nylon mesh filter, and the filtrate was centrifuged at 300 × *g*, 4°C for 5 min. Blood and spinal cord samples were then lysed with 1 ml of 1× RBC lysis buffer (eBioscience) for 5 min at 4°C, followed by the addition of 10 ml of FACS buffer (0.5 µM EDTA, 2% fetal bovine serum, and HBSS; pH 7.4). The cell suspension was centrifuged and the pellet was re-suspended in 10 ml of HBSS and centrifuged again. Live/dead cell staining was then performed by incubating samples with Ghost Dye Red 780 (1:1000, Tonbo Biosciences) for 30 min at 4°C. Samples were washed with 10 ml of FACS buffer, centrifuged, and the supernatant was discarded. Cells were then resuspended in FACS buffer and incubated with anti-mouse CD16/32 Fc blocking antibody (1:100 dilution; eBioscience, RRID:AB_467133) at 4°C for 20 min. Next, samples were washed with 10 mL of FACS buffer and 1 ml was partitioned for isotype control staining. Samples were then centrifuged and the pellets were incubated with a cocktail of the following antibodies: FITC-conjugated rat anti-mouse CD62L (1:100 dilution, Mel-14 clone, BioLegend, RRID:AB_313093), Pacific Blue-conjugated rat anti-mouse Ly-6G (1:200 dilution, 1A8 clone, BioLegend, RRID:AB_2251161), APC-conjugated rat anti-mouse CD11b (1:200 dilution, M1/70 clone, BioLegend, RRID:AB_312795), PE-conjugated rat anti-mouse CD45 (1:200 dilution, 30-F11 clone, Tonbo, RRID:AB_2621763), and PerCP/Cy5.5-conjugated rat anti-mouse Ly-6C (1:600 dilution, HK1.4 clone, BioLegend, RRID:AB_1659241). FITC-conjugated rat IgG2a, κ (1:100 dilution, BioLegend, RRID:AB_2736919) served as the isotype control for CD62L. After incubating with antibodies for 30 min at 4°C, samples were washed with FACS buffer, centrifuged, and the pellets were resuspended in 200 µl of FACs buffer. Flow cytometry was performed on the cell suspensions using a Fortessa flow cytometer (Becton Dickinson).

The data were analyzed using FlowJo software. At least 50,000 events were analyzed for blood samples, and all of the collected events were analyzed for spinal cord samples. Cells (low SSC, high FSC) were gated from debris (high SSC, low FSC) as previously described (Stirling and Yong, 2008). The geometric mean fluorescent intensity (G-MFI) values for CD62L were calculated to determine the overall presence of L-selectin on the surface of leukocytes. Since staining histograms for L-selectin were often skewed, we used G-MFI instead of arithmetic MFI to represent the data. This parameter better reflects the central tendency of skewed distributions. Isotype G-MFI were determined for each sample and subtracted from the reported G-MFI values. Total leukocyte and myeloid lineage subset counts from spinal cord samples were extrapolated to the total volume of cells to account for the volume of cells removed for isotype staining.

### sL-selectin ELISA

To determine whether diclofenac treatment induces L-selectin shedding, blood was obtained by cardiac puncture at 0, 2, 8, 24, and 48 h after intraperitoneal administration of this drug (1, 5, 10, 20, 40, or 60 mg/kg) immediately following SCI produced by dropping a 2 g weight from a distance of 5 cm. After centrifugation at 2000 × *g* for 20 min at RT, plasma was then collected for assessment using an ELISA kit (Quantikine, mouse sL-Selectin, R&D Systems) according to the manufacturer’s instructions. All incubations were conducted at RT and for 2 h unless otherwise indicated. A total of 100 µl of supernatants was first incubated with assay diluent buffer in a microplate. After rinsing, the preparation was then incubated in 100 µl of sL-selectin conjugate, followed by addition of 100 µl of substrate for 30 min. The reaction was then stopped by addition of stop solution. Optimal density was read at 450-nm wavelength with correction at 540 nm using a microplate reader (Invitrogen).

### Experimental design and statistical analyses

The primary goal of this study was to determine whether L-selectin contributes to inflammation and neurologic deficits following SCI. We employed L-selectin KO mice and also evaluated the effect of diclofenac, an NSAID that induces L-selectin shedding, on inflammation and neurologic recovery after SCI (Table 1). For all studies, only adult male mice were used to eliminate gender as a variable. Time points for collection of data and experimental endpoints were predetermined based on previous experience. Sample sizes were determined by our previous experience with BMS scoring and immunoblotting (Lin et al., 2007; Lee et al., 2011; Whetstone et al., 2017), and by power analyses for flow cytometry studies and sL-selectin assays (effect sizes were 500 G-MFI units and 550 ng/ml, respectively, based on our own preliminary studies). For all studies, we anticipated a ∼10% mortality rate, based on previous experience in our lab (Whetstone et al., 2017) and substantiated in Figure 10*I*, and we adjusted the samples sizes accordingly. To control for potential variation in the SCI surgery and behavioral analyses, all studies were conducted with the surgeon and observers blinded to the genotype and/or experimental condition. Simple or block randomization was used for all *in vivo* studies.

Statistical analyses were performed using GraphPad Prism (GraphPad Software). Flow cytometry was evaluated by unpaired two-tailed Student’s *t* tests or one-way ANOVA followed by Tukey’s *post hoc* test. ELISA data were evaluated by one-way ANOVA followed by a Dunnett’s *post hoc* test. A two-way, repeated measures ANOVA followed by Sidak’s multiple comparisons test was used to evaluate BMS scores. Comparison of two groups (foot faults, spared white matter, and immunoblots) was by an unpaired two-tailed Student’s *t* test. χ^2^ analysis was used to compare the number of animals stepping in the open field followed by a one-sided Fisher’s exact test. Comparison of two groups was performed by the Mann–Whitney test when at least one of the datasets failed the D’Agostino and Pearson omnibus normality test. Statistical significance was defined at *p* ≤ 0.05. Data are expressed as mean ± SEM.

### Data availability

All data are available from the authors.

## Results

### Functional recovery is improved in L-selectin KO mice

**Figure 1.**
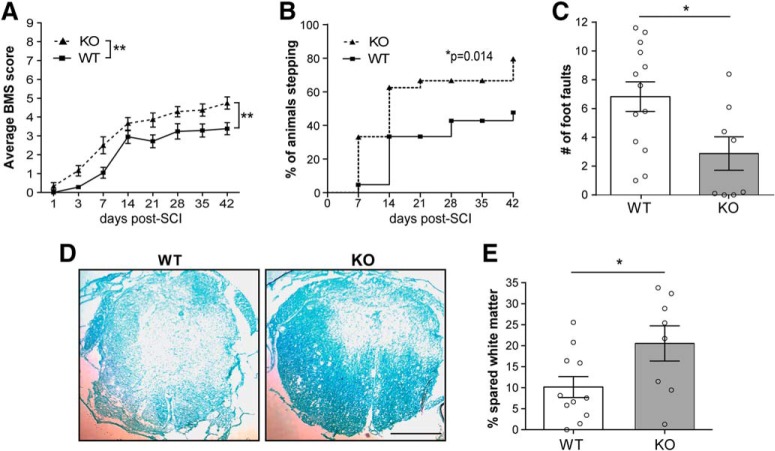
L-selectin KO mice show improved functional recovery and greater white matter sparing at 42 d after SCI. ***A***, Functional recovery, as measured by the BMS open field test, was improved in KO mice. *N* = 21 for WT and *N* = 24 for KO combined from two independent cohorts of mice. Two-way ANOVA (interaction *p* = 0.11; time *p* < 0.0001; genotype *p* = 0.006; *F*_(1,43)_ = 8.42). ***B***, Stepping was improved in KO mice compared to WT mice. *N* = 21 for WT and 24 for KO combined from two independent cohorts of mice. χ^2^ analysis followed by a one-sided Fisher’s exact test. *p = 0.029. ***C***, Performance on a grid, as measured by foot faults, was improved in the KO group. *N* = 13 for WT and *N* = 8 for KO. Two-tailed Student’s *t* test. *p = 0.023, *t*_(19)_ = 2.48. ***D***, Representative transverse sections at the lesion epicenter, stained with LFB, of injured WT and KO mice at 42 d post-SCI. Scale bar = 500 µm. ***E***, Greater spared white matter at the lesion epicenter, expressed as proportional area, was observed in the KO group. *N* = 11 for WT and *N* = 8 for KO. Two-tailed Student’s *t* test. *p = 0.038, *t*_(17)_ = 2.26.

To assess whether L-selectin is a determinant of recovery after SCI, we first compared locomotion of spinal cord-injured L-selectin KO mice with WT littermate controls using the BMS. L-selectin KO mice, subjected to SCI, showed a marked improvement in locomotor recovery compared to that of the WT group (*p* = 0.006; Fig. 1*A*). A subsequent secondary analysis revealed that 79.2% of KO animals achieved weight supported plantar stepping (BMS ≥ 4) compared to 47.6% of WT mice by 42 d post-SCI (*p* = 0.029; Fig. 1*B*). We also determined the number of foot-faults of mice crossing a wire grid, which reflects sensorimotor function and motor coordination (Schaar et al., 2010). Foot-faults were reduced by 57.9% in the KO relative to the WT group at 35 d post-SCI (*p* = 0.023; Fig. 1*C*).

Functional recovery has been previously associated with greater white matter sparing at the lesion epicenter (Kuhn and Wrathall, 1998). To determine whether L-selectin is associated with white matter damage, the percentage of spared white matter was compared between groups in transverse sections stained with LFB (Fig. 1*D*). Strikingly, the percentage of spared white matter at the lesion epicenter was 2.0-fold greater in the spinal cord-injured KO mice as compared to injured WT mice at 42 d post-SCI (*p* = 0.038; Fig. 1*E*).

### L-selectin is a determinant of oxidative stress in the acutely injured cord

**Figure 2.**
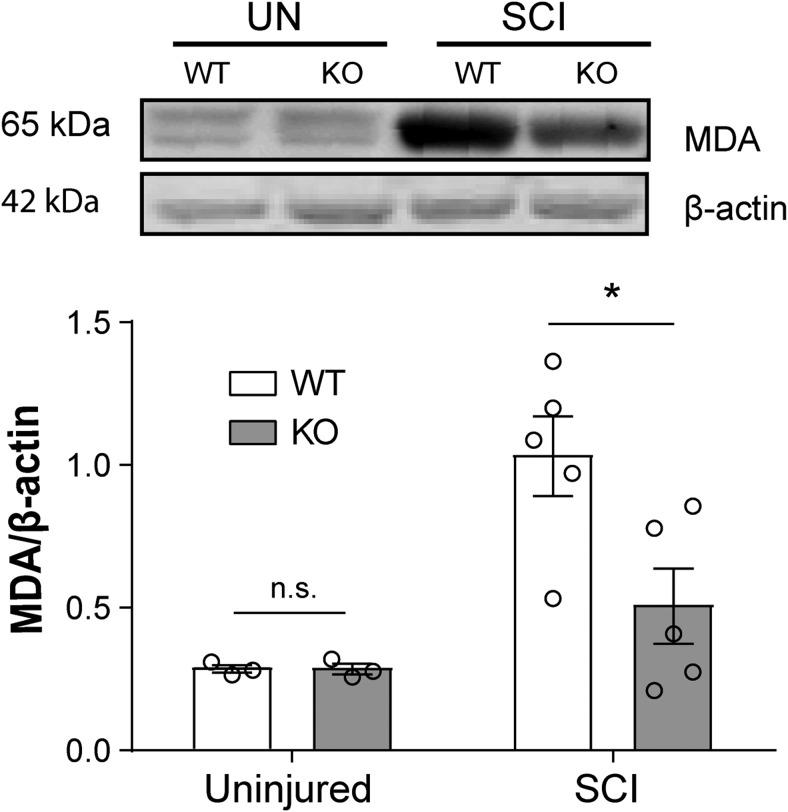
L-selectin is a determinant of early oxidative stress in the injured cord. Immunoblotting was performed on homogenates of uninjured (UN) and injured (SCI) cords from WT and L-selectin KO mice at 3 d post-SCI. All values were normalized to β-actin. By immunoblotting, MDA was reduced in L-selectin KO mice by 51% compared to WT mice after SCI (*p = 0.026, *t*_(8)_ = 2.73). *N* = 3/genotype for UN and *N* = 5/genotype for SCI. Unpaired two-tailed Student’s *t* test. For UN, *p* = 0.98, *t*_(4)_ = 0.02.

Newly recruited neutrophils and monocytes/macrophages from the blood promote oxidative stress in the acutely injured cord (Lee et al., 2011). Since L-selectin is expressed on blood neutrophils and monocytes, we examined the impact of the absence of L-selectin on oxidative stress. Immunoblotting verified that MDA, a product of lipid peroxidation (Liu et al., 2001) and marker for acute oxidative stress (Esterbauer et al., 1991; Azbill et al., 1997; Hichor et al., 2018), was similarly present at very low levels in the uninjured spinal cords of mice of both genotypes (*p* = 0.98; Fig. 2). There was a 51% reduction in MDA in injured L-selectin KO mice compared to injured WT mice (*p* = 0.026, 3 d post-SCI).

### L-selectin is dynamically regulated on myeloid cells following injury

**Figure 3.**
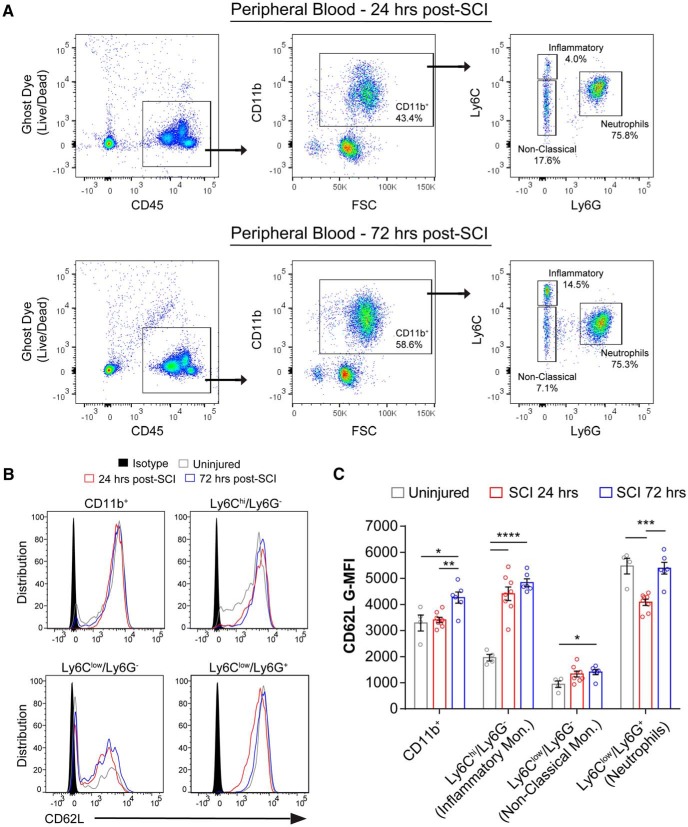
L-selectin is dynamically regulated after SCI. ***A***, Flow cytometry gating of leukocytes and leukocyte subsets in the peripheral blood at 24 and 72 h post-SCI. ***B***, Representative flow cytometry histograms for CD62L staining of myeloid cells in the peripheral blood from uninjured mice (gray line) and injured mice at 24 h (red line) and 72 h (blue line) post-SCI. Representative isotype staining is shown in solid black. ***C***, L-selectin (CD62L) levels on myeloid lineage cells in the peripheral blood from uninjured WT mice, and injured WT mice at 24 and 72 h post-SCI. *N* = 4 for UN, *N* = 8 for 24 h, and *N* = 6 for 72 h. One-way ANOVA with Tukey’s *post hoc* test (*p* = 0.004, <0.0001, 0.046, and 0.0001 and *F*_(2,15)_ = 7.97, 35.8, 3.80, and 17.7, respectively). *p < 0.05, **p < 0.01, ***p < 0.001, ****p < 0.0001.

**Figure 4.**
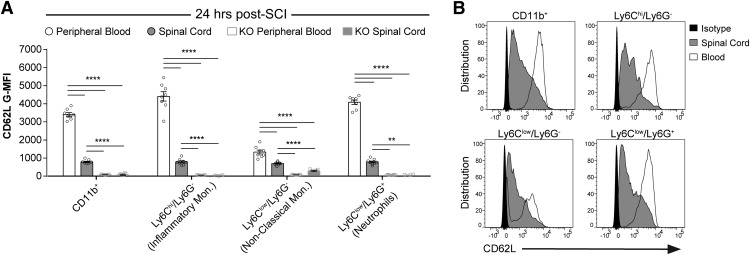
L-selectin is partially shed during infiltration into the spinal cord. ***A***, L-selectin levels (G-MFI) on circulating and infiltrated myeloid lineage cells from WT and KO mice at 24 h post-SCI. Background (isotype) values for CD62L were subtracted from each datapoint so that the *y*-axis represents the specific signal. Autofluorescence resulted in a faint signal in non-classical monocytes in the spinal cord but not in the blood of L-selectin KO mice. This was likely due to phagocytosed debris. *N* = 8/group. One-way ANOVA with Tukey’s *post hoc* test (*p* < 0.0001 for each cell type and *F*_(3,27)_ = 5.49, 7.50, 4.71, and 7.92, respectively). **p < 0.01, ****p < 0.0001. ***B***, Representative flow cytometry histograms for CD62L staining in leukocyte populations from the blood (white) and spinal cord (gray) of WT mice at 24 h post-SCI. Representative isotype staining is shown in solid black.

In light of our findings with L-selectin KO mice, we employed flow cytometry to quantify the expression of L-selectin on circulating and spinal cord-infiltrated leukocytes after SCI. Peripheral blood leukocytes from uninjured WT mice were also analyzed to establish baseline L-selectin levels. Because of their prominent recruitment during the acute phase of SCI (Beck et al., 2010), we focused on myeloid cells (CD45^+^/CD11b^+^). We identified leukocytes by gating for live (Ghost Dye^-^) CD45^+^ cells, then gated for myeloid cells (CD11b^+^) and subdivided them into neutrophils (Ly6C^low^/Ly6G^+^), inflammatory/classical monocytes (Ly6C^hi^/L6G^-^), and patrolling/non-classical monocytes (Ly6C^low^/Ly6G^-^; Fig. 3*A*). For the monocyte populations in peripheral blood, SCI resulted in over 2-fold greater levels (G-MFI) of L-selectin (CD62L) on Ly6C^hi^/L6G^-^ cells at 24 and 72 h post-SCI (*p* < 0.0001) and an increase of 49.4% on Ly6C^low^/L6G^-^ cells at 72 h post-SCI (*p* = 0.048) compared to uninjured mice (Fig. 3*B*,*C*). In contrast, L-selectin decreased by 25.3% on neutrophils (Ly6C^low^/Ly6G^+^ cells) at 24 h post-SCI (*p* = 0.007) but returned to baseline (uninjured) values by 72 h post-SCI. Leukocytes that accumulated in the spinal cord parenchyma at 24 h post-SCI exhibited two to 5-fold reduced levels of L-selectin on the myeloid subtypes relative to levels on the corresponding cells in the blood (Fig. 4*A*); however, considerable L-selectin remained on the infiltrated cells for all subtypes, as established by comparison with corresponding cells in KO mice (Fig. 4*A*) and isotype controls (Fig. 4*B*). These data demonstrate that L-selectin is present in varying degrees on all circulating myeloid lineages and is dynamically regulated after SCI. Furthermore, considerable L-selectin is retained on infiltrated myeloid cells, where it could potentially be involved in post-recruitment adhesive or signaling events.

### L-selectin deficiency transiently reduces myeloid cell accumulation in the spinal cord

**Figure 5.**
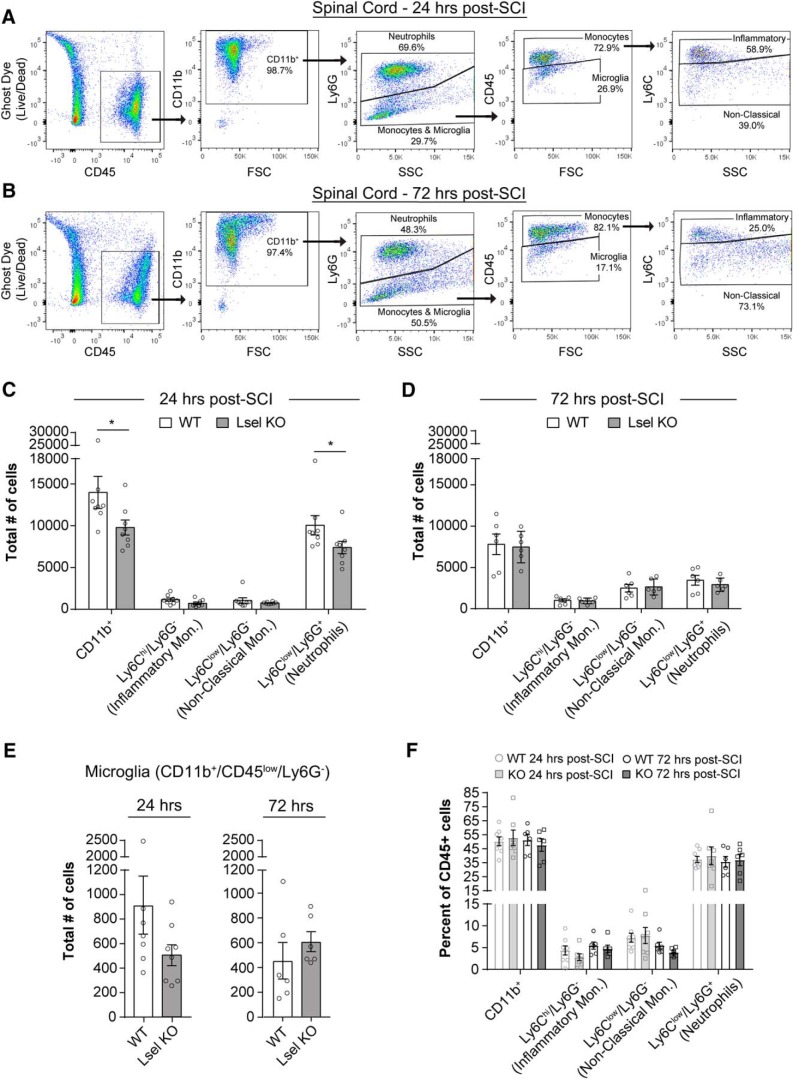
Leukocyte accumulation is transiently reduced in KO mice compared to WT mice after SCI. ***A***, ***B***, Flow cytometry gating of leukocytes and leukocyte subsets in the injured spinal cord at ***A***, 24 h and ***B***, 72 h post-SCI. ***C***, Accumulation of myeloid cells (CD11b^+^) and neutrophils (Ly6C^low^/Ly6G^+^), as determined by flow cytometry, was reduced in L-selectin KO compared to WT mice at 24 h post-SCI (*p = 0.028 and 0.038, respectively). There were no differences in infiltrating inflammatory (Ly6C^hi^/Ly6G^-^, *p* = 0.10) or non-classical monocyte subsets (Ly6C^low^/Ly6G^-^, *p* = 0.72) between WT and KO mice. *N* = 7 for WT and *N* = 8 for KO. Mann–Whitney test. ***D***, There was no difference in the accumulation of any leukocyte subtype at 72 h post-SCI. *N* = 6/group. Unpaired two-tailed Student’s *t* tests. *p* = 0.82, 0.81, 0.81, and 0.45 and *t*_(10)_ = 0.23, 0.25, 0.24, and 0.79, respectively. ***E***, There were no differences in microglia (CD11b^+^/CD45^low^/Ly6G^-^) between WT and KO mice at 24 or 72 h post-SCI (*p* = 0.10 and 0.38, respectively). ***F***, Prevalence of myeloid lineage subsets in the peripheral blood was similar between WT and KO mice at 24 and 72 h post-SCI. *N* = 7–8/genotype. Unpaired two-tailed Student’s *t* tests. No differences were detected (*p* = 0.68, 0.25, 0.80, and 0.70 and *t*_(13)_ = 0.42, 1.19, 0.25, and 0.40, respectively).

As reviewed in the Introduction, L-selectin has been implicated in the recruitment of myeloid cells in multiple inflammatory settings. We therefore asked whether the lack of L-selectin could reduce trafficking of myeloid cells into the injured cord. We compared the number of leukocytes in injured spinal cords of L-selectin KO and WT mice. The accumulation of leukocytes in a particular tissue reflects recruitment from the blood and their turnover in the tissue. Using flow cytometry, we first determined the total number of infiltrated myeloid lineage cells (CD45^+^/CD11b^+^). We then used Ly6C and Ly6G antibodies to discriminate neutrophils and monocyte/microglia subtypes (Fig. 5*A*,*B*), and further subdivided the monocyte and resident microglia populations based on CD45 levels. Few CD11b^+^ cells were detected in the spinal cords of uninjured WT mice (650 ± 103 cells) relative to injured WT mice at 24 h post-SCI (13970 ± 1920 cells). At 24 h post-SCI, very few infiltrated CD11b^+^ cells co-labeled for T-cells (213 ± 60 TCRβ^+^ cells) or B-cells (357 ± 113 B220^+^ cells), confirming that CD11b^+^ cells were predominantly myeloid cells. Furthermore, no differences in CD45^+^/CD11b^-^ cells (lymphocytes) were observed in the spinal cords of uninjured versus injured WT mice at 24 h post-SCI (485 ± 186 vs 532 ± 178 cells, *p* = 0.87). At 24 h post-SCI, we observed reductions of 29.9% and 26.4% of CD11b^+^ cells (*p* = 0.028) and Ly6C^low^/L6G^+^ cells (*p* = 0.038), respectively, but no significant effects on Ly6C^hi^/L6G^-^ cells (*p* = 0.10) or Ly6C^low^/Ly6G^-^ cells (*p* = 0.72) in the spinal cords of L-selectin KO mice compared to WTs (Fig. 5*A*,*C*). Clearly, the decrease in CD11b^+^ cells was mainly attributable to the Ly6C^low^/L6G^+^ cells (neutrophils), which comprised ∼72% of this population. The contributions of L-selectin to myeloid cell and neutrophil accumulation were limited in duration as there were no differences in these populations between KO and WT mice at 72 h post-SCI (Fig. 5*B*,*D*). No differences in the total number of CD11b^+^/CD45^low^/L6G^-^ cells (microglia) were observed between WT and KO mice at 24 and 72 h post-SCI (*p* = 0.10 and 0.38, respectively; Fig. 5*E*). No differences were observed in the proportion of CD11b^+^ cells and myeloid lineage subsets among total CD45^+^ leukocytes in the peripheral blood of WT and KO mice at 24 and 72 h post-SCI (*p* > 0.25 for all subtypes; Fig. 5*F*). In summary, we found that SCI was associated with a massive influx of myeloid cells (21-fold increase) into the spinal cord and the absence of L-selectin resulted in a partial reduction in the accumulation of neutrophils at 24 h, which was not sustained through 72 h.

### NSAIDS differentially induce L-selectin shedding on murine leukocytes

**Figure 6.**
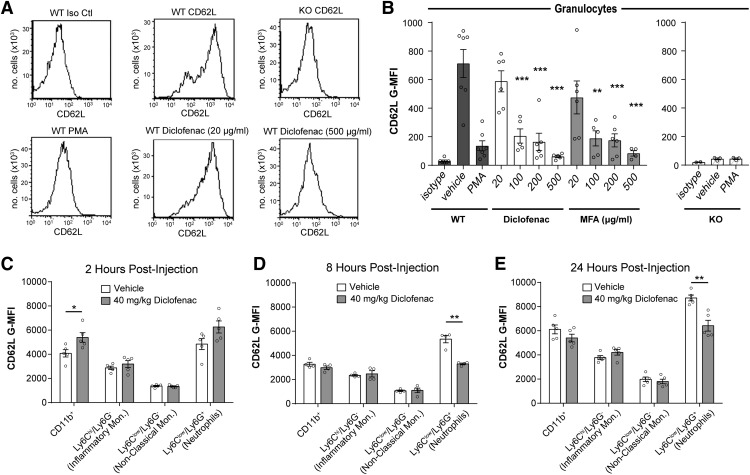
NSAIDs induce L-selectin shedding. ***A***, CD62L flow cytometry histograms for WT or L-selectin KO granulocytes treated with PMA or diclofenac (20 or 500 μg/ml) and stained with an isotype control antibody (Iso Ctl) or a CD62L antibody. ***B***, CD62L expression (G-MFI) on WT murine granulocytes was reduced relative to vehicle (*N* = 7) by treatment with diclofenac (*N* = 5–6) and MFA (*N* = 4–6). *N* = 3/group for KOs. Data were obtained from seven independent experiments. Cells were pooled from three mice for each experiment (21 total mice). One-way ANOVA with Dunnett’s *post hoc* test. For diclofenac, *p* < 0.0001 and *F*_(4,24)_ = 16.2. For MFA, *p* < 0.0001 and *F*_(4,23)_ = 10.0. *p < 0.05, **p < 0.01, ***p < 0.001. ***C–E***, CD62L expression (G-MFI) was reduced on neutrophils (Ly6C^low^/Ly6G^+^) in uninjured mice receiving diclofenac relative to vehicle control at 8 and 24 h after injection. *N* = 4/group at 8 h and *N* = 5/group at 2 and 24 h. Unpaired two-tailed Student’s *t* tests. *p = 0.031, 0.0006, and 0.002, and *t*_(8)_ = 2.61, *t*_(6)_ = 6.64, and *t*_(8)_ = 4.52, respectively.

As an alternative to the genetic ablation of L-selectin, we sought a pharmacologic means to lower the cell surface levels of this receptor. A major mechanism for down-modulating L-selectin is through cleavage of the membrane proximal domain of L-selectin by cell surface metalloproteinases, such as ADAM17, causing the release (shedding) of the ligand-binding ectodomain into solution (Li et al., 2006). Certain NSAIDs can induce L-selectin shedding in human leukocytes *in vitro* and *in vivo* (Díaz-González et al., 1995), which led us to investigate whether an NSAID could be used to down-modulate L-selectin levels on myeloid cells during SCI and produce benefit. We first performed *in vitro* experiments to test NSAIDs for their ability to induce L-selectin shedding from murine myeloid cells. We evaluated diclofenac and MFA, which were shown to produce the most L-selectin shedding in human leukocytes (Gómez-Gaviro et al., 2002). Murine granulocytes (predominantly neutrophils), exposed *in vitro* to either diclofenac or MFA, exhibited a dose-dependent reduction in the level of L-selectin on the cell surface (Fig. 6*A*,*B*).

The *in vitro* effects of a drug on particular leukocytes do not necessarily predict its *in vivo* activities with critical factors being the pharmacokinetics of the drug and the turnover and anatomic compartmentalization of leukocytes. To determine whether diclofenac induced the loss of cell surface L-selectin *in vivo*, uninjured WT mice were administered diclofenac (40 mg/kg, i.p.) and peripheral blood leukocytes were evaluated 2, 8, or 24 h in separate cohorts of mice for each time point. At 2 h post-injection, increased L-selectin was detected on peripheral blood CD11b^+^ cells from diclofenac-treated mice compared to saline-treated mice (32.2% increase, *p* = 0.03; Fig. 6*C*), possibly due to mobilization of myeloid cells from the bone marrow. At 8 h post-injection, there was a 38.3% reduction of L-selectin on Ly6C^low^/Ly6G^+^ cells (*p* = 0.0006; Fig. 6*D*) and a reduction of 26.3% at 24 h (*p* = 0.002; Fig. 6*E*).

### Diclofenac improves long-term functional recovery

**Figure 7.**
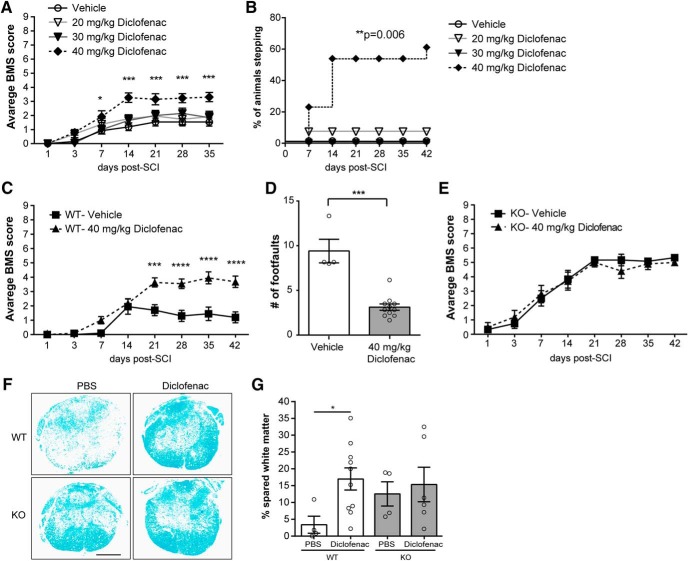
Long-term functional recovery and white matter sparing are improved in spinal cord-injured WT mice treated with diclofenac immediately after injury. ***A***, Spinal cord-injured mice treated with 40 mg/kg diclofenac showed improved recovery starting at 7 d post-SCI as determined by the BMS open field test. *N* = 10 for 30 mg/kg diclofenac, and *N* = 13 for all other groups. Repeated measures two-way ANOVA with Tukey’s *post hoc* test (interaction *p* < 0.001; time *p* < 0.001; treatment *p* = 0.003 and *F*_(3,45)_ = 5.50). ***p < 0.0001 for vehicle versus 40 mg/kg diclofenac. No differences were observed between vehicle and 20 or 30 mg/kg diclofenac. ***B***, Stepping was improved in 40 mg/kg diclofenac-treated mice compared to mice receiving vehicle control. *N* = 10 for 30 mg/kg diclofenac, and *N* = 13 for all other groups. χ^2^ analysis followed by a one-sided Fisher’s exact test. **p = 0.006 for vehicle versus 40 mg/kg. *p* = 0.006 for 20 versus 40 mg/kg and *p* = 0.003 for 30 versus 40 mg/kg. ***C***, Spinal cord-injured WT mice treated with 40 mg/kg diclofenac showed improved recovery compared to vehicle controls. *N* = 10 for vehicle and *N* = 11 for diclofenac. Repeated measures two-way ANOVA with Sidak’s *post hoc* test (interaction *p* < 0.001, time *p* < 0.0001, treatment *p* = 0.0004, and *F*_(1,19)_ = 18.8). ***p < 0.001, ****p < 0.0001. ***D***, The number of foot faults was reduced in diclofenac-treated mice. *N* = 4 for PBS and *N* = 11 for diclofenac. Mann–Whitney test. ***p = 0.0007. ***E***, Functional recovery was similar between L-selectin KO mice treated with diclofenac or vehicle (PBS). *N* = 6 for PBS and *N* = 5 for diclofenac. Repeated measures two-way ANOVA (interaction *p* = 0.54, time *p* < 0.0001, treatment *p* = 0.85 and *F*_(1,9)_ = 0.04). ***F***, Representative images of the lesion epicenter, stained with LFB, in mice treated with vehicle (PBS) or diclofenac. Scale bar = 500 μm. ***G***, Spared white matter was increased in WT mice, but not L-selectin KO mice, treated with diclofenac compared to vehicle (PBS) at 42 d post-SCI. Data are expressed as proportional area. *N* = 4 for PBS-WT, *N* = 10 for diclofenac-WT, *N* = 4 for PBS-KO, and *N* = 6 for diclofenac-KO. Unpaired two-tailed Student’s *t* test. *p = 0.030 and *t*_(12)_ = 2.46. For KO, *p* = 0.70 and *t*_(8)_ = 0.40.

Having verified that diclofenac down-modulates L-selectin levels on myeloid cells *in vitro* and *in vivo*, we asked whether this drug could promote long-term recovery after SCI. Mice were treated with a single dose (20, 30, or 40 mg/kg) of diclofenac or vehicle (PBS) immediately after SCI, and neurologic function was assessed using the BMS open field test. We found considerable improvement in locomotor recovery at the highest dose, but not at the lower doses. Enhanced recovery was apparent by 7 d post-SCI and persisted throughout the remainder of the testing period (Fig. 7*A*). A subsequent secondary analysis revealed that by day 35, 61.5% of WT mice treated with 40 mg/kg diclofenac were occasionally stepping as opposed to 0.0% of PBS-treated mice (*p* = 0.002; Fig. 7*B*).

Diclofenac as an NSAID with a multiplicity of anti-inflammatory activities (Scholer et al., 1986) could provide benefit independently of its effects on L-selectin. To address this issue, we compared neurologic recovery and long-term white matter sparing in L-selectin KO mice subjected to SCI and treated with 40 mg/kg diclofenac or vehicle. As a positive control, we first examined diclofenac in WTs and again observed improved long-term neurologic recovery (Fig. 7*C*,*D*). We then examined diclofenac- and vehicle-treated L-selectin KO mice and found no differences based on the BMS scale (Fig. 7*E*). The percentage of spared white matter was also quantified at the lesion epicenter (Fig. 7*F*,*G*). Diclofenac-treated WT mice (40 mg/kg) showed 5-fold greater white matter sparing compared to PBS-treated WT mice (*p* = 0.030), whereas there was no difference in white matter sparing in KO mice treated with diclofenac versus PBS (*p* = 0.70). Thus, while diclofenac promoted recovery and neuroprotection in spinal cord-injured WTs, it provided no added long-term benefit in the absence of L-selectin.

### Diclofenac induces loss of L-selectin from specific leukocyte subsets but does not affect leukocyte accumulation in the injured spinal cord

**Figure 8.**
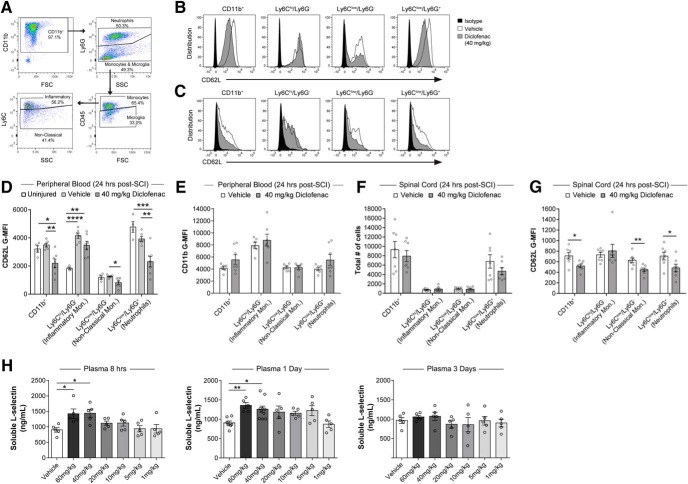
Diclofenac treatment reduces L-selectin on peripheral blood and infiltrated leukocytes but does not alter accumulation in the injured spinal cord. ***A***, Flow cytometry gating for infiltrated myeloid cells and myeloid subsets. ***B***, ***C***, Representative flow cytometry histograms for CD62L staining in leukocyte populations from the blood (***B***) or spinal cord (***C***) of vehicle-treated (white) and diclofenac-treated (gray) mice at 24 h post-SCI. Representative isotype staining is shown in black. ***D***, Flow cytometry analysis for CD62L expression on peripheral blood leukocytes from uninjured mice and injured mice treated with diclofenac or vehicle (PBS). Diclofenac induced a reduction of L-selectin on neutrophils and non-classical monocytes (*p* = 0.005 and 0.02, respectively). *N* = 4/uninjured and *N* = 6–7/SCI/treatment. One-way ANOVA with Tukey’s *post hoc* test (*p* = 0.004, <0.0001, 0.019, and 0.0004 and *F*_(2,14)_ = 8.47, 20.3, 5.30, and 14.8, respectively). *p < 0.05, **p < 0.01, ***p < 0.001. ***E***, Flow cytometry analysis for CD11b levels on peripheral blood leukocytes from spinal cord-injured mice treated with diclofenac or vehicle (PBS). There were no differences in CD11b levels on myeloid cells or myeloid lineage subsets. *N* = 6–7/treatment. Unpaired two-tailed Student’s *t* tests. *p* = 0.15, 0.47, 0.99, and 0.16 and *t*_(11)_ = 1.5, 0.75, 0.01, and 1.5, respectively. ***F***, There were no differences in the accumulation of total myeloid cells (CD11b^+^) or any myeloid lineage subset in spinal cords of diclofenac-treated mice compared to vehicle-treated mice at 24 h post-SCI. *N* = 7/treatment. Unpaired two-tailed Student’s *t* tests. *p* = 0.37, 0.53, 0.92, and 0.23 and *t*_(12)_ = 0.66, 0.64, 0.12, and 1.27, respectively. ***G***, Flow cytometry analysis demonstrated loss of L-selectin on total leukocytes (CD45^+^, *p* = 0.020 and *t*_(12)_ = 2.67), total myeloid cells (CD11b^+^, *p = 0.020 and *t*_(12)_ = 2.69), non-classical monocytes (Ly6C^low^/Ly6G^-^, **p = 0.008 and *t*_(12)_ = 3.16), and neutrophils (Ly6C^low^/Ly6G^+^, *p = 0.049 and *t*_(12)_ = 2.20) in the spinal cord of diclofenac-treated versus vehicle-treated mice at 24 h post-SCI. There was no effect on L-selectin on inflammatory monocytes (Ly6C^hi^/Ly6G^-^, *p* = 0.59 and *t*_(12)_ = 0.56). *N* = 7/treatment. Unpaired two-tailed Student’s *t* tests. ***H***, ELISA for sL-selectin in the peripheral blood at 8, 24, and 72 h post-SCI in mice receiving a vehicle (PBS) control injection or 1–60 mg/kg of diclofenac. Increased sL-selectin was observed at 8 and 24 h, but not 72 h, post-SCI in mice receiving 40 and 60 mg/kg diclofenac. *N* = 5/group for 8 and 72 h; *N* = 5/group at 24 h except for vehicle (*N* = 7) and 40 mg/kg diclofenac (*N* = 10). One-way ANOVA followed by Dunnett’s *post hoc* test (*p* = 0.005, 0.006, and 0.71 and *F*_(6,28)_ = 4.05, *F*_(6,35)_ = 3.72, and *F*_(6,28)_ = 0.63, respectively). *p < 0.05, **p < 0.01.

We next determined whether diclofenac treatment affected leukocyte accumulation after SCI. A single dose of diclofenac (40 mg/kg) or vehicle (PBS) was given immediately after SCI in WT mice. Peripheral blood and spinal cords were harvested 24 h later, and flow cytometry was performed to determine the levels of L-selectin as well as the accumulation of leukocyte subsets in the cord (Fig. 8*A*). Peripheral blood was also acquired from uninjured WT mice to compare basal expression levels of L-selectin in circulating myeloid lineages. Consistent with the results above (Fig. 3*B*), at 24 h post-SCI, injury itself (with saline injection) resulted in over 2-fold greater levels of L-selectin on Ly6C^hi^/Ly6G^-^ cells (*p* = 0.005; Fig. 8*B*,*D*) in blood. At 24 h post-SCI, diclofenac reduced L-selectin on CD11b^+^ cells (38.2% reduction, *p* = 0.004), Ly6C^low^/Ly6G^-^ cells (34.6% reduction, *p* = 0.022), and Ly6C^low^/Ly6G^+^ cells (41.1% reduction, *p* = 0.005), but not on Ly6C^hi^/Ly6G^-^ cells (*p* = 0.12) in blood of injured mice compared to vehicle-treated controls (Fig. 8*B*,*D*). Importantly, diclofenac did not activate myeloid cells in the blood (24 h post-SCI), as reflected by no change in CD11b levels (Fig. 8*E*). Thus, the actions of diclofenac do not appear to be attributable to a generalized activation of these cells.

No differences were apparent in the total numbers of CD11b^+^ cells or myeloid lineage subsets in the spinal cords of diclofenac-treated mice at 24 h post-SCI compared to mice receiving the vehicle control (Fig. 8*F*). However, diclofenac reduced the level of L-selectin on infiltrated CD11b^+^ cells (26.7% loss, *p* = 0.020; Fig. 8*C*,*G*), Ly6C^low^/Ly6G^-^ cells (28.8% loss, *p* = 0.008), and Ly6C^low^/Ly6G^+^ cells (31.8% loss, *p* = 0.049). There was no effect on Ly6C^hi^/Ly6G^-^ cells (*p* = 0.59). Thus, while diclofenac did not alter leukocyte accumulation in the injured cord, L-selectin was reduced on the same myeloid populations (i.e., neutrophils and non-classical monocytes) in both the blood and spinal cord of injured animals. To substantiate that diclofenac treatment resulted in shedding of L-selectin from leukocytes, we measured sL-selectin in blood plasma by ELISA. We employed a single dose of diclofenac at increasing doses (1, 5, 10, 20, 40, and 60 mg/kg) immediately following SCI. Elevated sL-selectin (above vehicle background) was detected at 8 and 24 h post-SCI with 40 and 60 mg/kg of diclofenac but not at the lower doses (Fig. 8*H*). The elevation did not persist at 72 h post-injection. Importantly, the high concentration of diclofenac required to induce L-selectin shedding paralleled the high concentration required to improve long-term neurologic recovery (Fig. 7*A*).

### Diclofenac reduces acute oxidative stress after SCI

**Figure 9.**
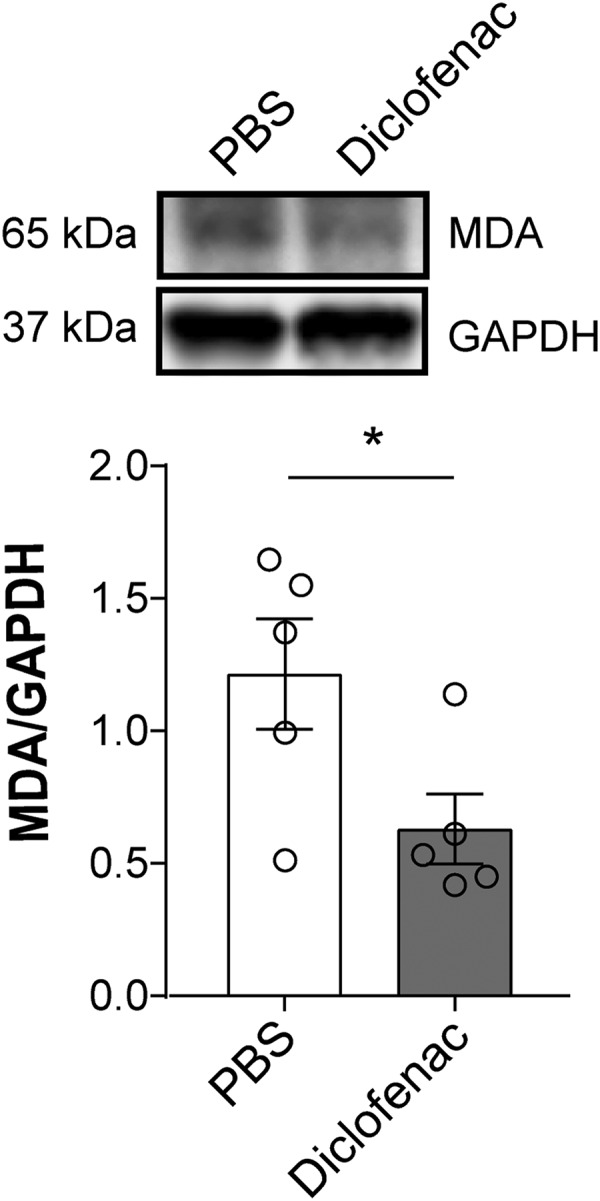
Diclofenac treatment reduces acute oxidative stress at 72 h post-SCI. By immunoblotting, MDA was reduced by 48.1% in diclofenac-treated versus vehicle-treated WT mice after SCI (*p = 0.045, *t*_(8)_ = 2.37). *N* = 5/treatment. Unpaired two-tailed Student’s *t* test. All values were normalized to GAPDH.

Antibody-induced ligation of L-selectin has been shown to induce or potentiate the production of ROS by neutrophils (Crockett-Torabi et al., 1995). Therefore, the reduced level of L-selectin on these cells following diclofenac treatment could potentially mitigate oxidative stress in the acutely injured spinal cord. We therefore examined MDA levels in the injured spinal cord at 72 h post-SCI in mice receiving diclofenac (40 mg/kg) or vehicle (PBS) immediately following SCI. There was a 48.1% reduction in MDA between diclofenac and vehicle-treated mice (*p* = 0.045; Fig. 9).

### Diclofenac improves recovery when delivered within 3 h after SCI

**Figure 10.**
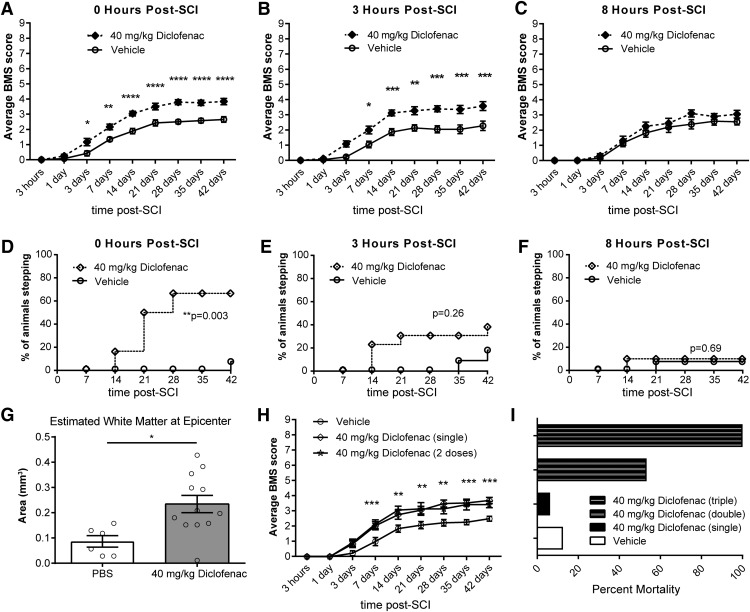
Diclofenac treatment improves long-term recovery when delayed for 3 h, but not 8 h, post-SCI. ***A***, BMS scores demonstrated improved neurologic recovery when diclofenac was delivered immediately following injury. *N* = 13 for vehicle and *N* = 12 for diclofenac. Two-way ANOVA with Sidak’s *post hoc* test (interaction *p* < 0.0001, time *p* < 0.0001, treatment *p* < 0.0001 and *F*_(1,23)_ = 26.0). *p < 0.05, **p < 0.01, ***p < 0.001, ****p < 0.0001. ***B***, Recovery was also improved when administration of diclofenac was delayed to 3 h post-SCI. *N* = 11 for vehicle and *N* = 13 for diclofenac. Two-way ANOVA with Sidak’s *post hoc* test (interaction *p* < 0.0001, time *p* < 0.0001, treatment *p* = 0.0005 and *F*_(1,22)_ = 17.0). *p < 0.05, **p < 0.01, ***p < 0.001. ***C***, No improvement was observed when diclofenac was delayed to 8 h post-SCI. *N* = 13 for vehicle and *N* = 10 for diclofenac. Two-way ANOVA with Sidak’s *post hoc* test (interaction *p* = 0.42, time *p* < 0.001, treatment *p* = 0.23 and *F*_(1,21)_ = 1.51). ***D***, Improved stepping ability was observed when diclofenac was delivered immediately after injury. χ^2^ analysis followed by a one-sided Fisher's exact test. *N* = 13 for vehicle and *N* = 12 for diclofenac. **p = 0.003. ***E***, No benefit for stepping was observed when diclofenac was delivered 3 h after injury. χ^2^ analysis followed by a one-sided Fisher’s exact test. *N* = 11 for vehicle and *N* = 13 for diclofenac. *p = 0.26. ***F***, No benefit for stepping was observed with an 8-h delay of diclofenac administration. χ^2^ analysis followed by a one-sided Fisher’s exact test. *N* = 13 for vehicle and *N* = 10 for diclofenac. *p* = 0.69. ***G***, Spared white matter at the lesion epicenter was increased at 42 d post-SCI in WT mice treated with diclofenac at 3 h post-SCI compared to the time-matched vehicle (PBS) control group. Data are expressed as total area. *N* = 6 for PBS and *N* = 12 for diclofenac. Two-tailed Student’s *t* test. *p = 0.011, *t*_(16)_ = 2.87. ***H***, No additional benefit in BMS score was achieved by administering a second dose of diclofenac. *N* = 13 for vehicle, *N* = 15 for single dose, and *N* = 7 for two doses. Two-way ANOVA with Tukey’s *post hoc* test (interaction *p* < 0.001, time *p* < 0.001, treatment *p* = 0.002 and *F*_(2,32)_ = 8.04). *p* > 0.55 for single versus two doses for all timepoints. *p < 0.05, **p < 0.01, ***p < 0.001. ***I***, Multiple doses of diclofenac resulted in higher mortality rates.

To determine the window for the beneficial effects of diclofenac, a single dose of diclofenac (40 mg/kg, i.p.) was administered at 0, 3, or 8 h following SCI. Again, we found that immediate delivery of diclofenac improved long-term neurologic recovery of hindlimb function, as seen by greater BMS scores within the first 7 d post-SCI compared to vehicle-treated mice (Fig. 10*A*). Stepping was also improved with 66.7% of diclofenac-treated mice stepping at 42 d post-SCI compared to 7.7% of vehicle-treated mice (*p* = 0.003; Fig. 10*D*). When administration of diclofenac was delayed for 3 h, improved neurologic recovery was evident starting at 7 d post-SCI compared to time-matched vehicle control mice (Fig. 10*B*). Although stepping was not improved (38.5% vs 18.2%, *p* = 0.26; Fig. 10*E*; Table 2), white matter sparing was 2.3-fold greater in mice receiving diclofenac at 3 h post-SCI compared to vehicle-treated mice (*p* = 0.011; Fig. 10*G*). There was no long-term benefit in BMS scores or stepping when diclofenac treatment was delayed to 8 h post-SCI (Fig. 10*C*,*F*).

**Table 1.** Number of animals

	WT (*N*)	KO (*N*)
Long-term studies		
SCI	21	24
BMS	21	24
Grid-walk	13	8
White matter sparing	11	8
Acute studies		
Uninjured	7	7
SCI	19	19
Immunoblotting	8	8
Flow cytometry	18	18
	Vehicle (*N*)	Diclofenac (*N*)
Long-term studies		
SCI (WT)	75	122
SCI (KO)	6	5
BMS	79	109
Grid walk	4	11
White matter sparing	14	28
Mortality (single dose)	2	1
Mortality (two doses)	N/A	8
Mortality (three doses)	N/A	9
Acute studies		
*In vitro*	21 (pooled)	
Uninjured	18	18
SCI	29	107
Flow cytometry	25	25
ELISA	17	95
Immunoblotting	5	5

**Table 2.** Categorical analysis of BMS scoring

	Vehicle (3 h post-SCI, *N* = 11)			Diclofenac (3 h post-SCI, *N* = 13)		
BMS category	1 d	7 d	42 d	1 d	7 d	42 d
0	100% (11)	27.3% (3)		100% (13)	7.7% (1)	
1		45.5% (5)	27.3% (3)		38.5% (5)	
2		27.3% (3)	45.5% (5)		15.4% (2)	30.8% (4)
3			9.1% (1)		38.5% (5)	30.8% (4)
4			18.2% (2)			7.7% (1)
5						30.8% (4)

To determine whether multiple doses of diclofenac conferred additional benefit, we randomly grouped mice into three cohorts and delivered diclofenac (40 mg/kg) at 0 h (single dose), 0 and 24 h (two doses), or 0, 24, and 48 h (three doses) post-SCI. Long-term neurologic benefit was observed in the single and two-dose groups with improved BMS scores starting at 7 d post-SCI compared to the vehicle-treated group (Fig. 10*H*). No additional benefit was observed for two doses compared to a single dose of diclofenac. BMS scores could not be determined for mice receiving three doses of diclofenac due to a high rate of morbidity and mortality. While a single dose of diclofenac exhibited a similar mortality rate to vehicle control injections, there was increased mortality for the double and triple dose regimens (Fig. 10).

## Discussion

The mechanisms underlying secondary pathogenesis, including the pathogenic activities of myeloid cells, after SCI are not fully understood, thereby limiting the development of clinical therapies. This is the first study to demonstrate the involvement of L-selectin in acute secondary pathogenesis in a murine model of SCI. We show that the genetic ablation of L-selectin markedly improves long-term neurologic recovery and white matter sparing. Pursuing a pharmacologic approach to reduce L-selectin, we demonstrate that the NSAID, diclofenac, induces partial L-selectin shedding from mouse myeloid cells *in vivo* and improves long-term neurologic outcomes when administered up to 3 h following injury. Our findings provide support for L-selectin shedding and the subsequent reduction of L-selectin-dependent activities other than myeloid cell recruitment, as an important anti-inflammatory activity of diclofenac during SCI.

Past studies have achieved success in limiting inflammation and secondary damage by targeting specific adhesion molecules (such as P-selectin, CD11d/CD18, and ICAM-1) and chemoattractants/chemokines involved in the migration of peripheral leukocytes into the spinal cord (Hamada et al., 1996; Taoka et al., 1997; Gonzalez et al., 2003; Gris et al., 2004; Popovich and Longbrake, 2008; Saiwai et al., 2010). In the present study, we have investigated L-selectin, which heretofore has not been considered in the context of SCI. A substantial body of evidence has established that L-selectin functions as adhesion/signaling molecule on myeloid cells and participates in their recruitment and activation at inflammatory sites (Lewinsohn et al., 1987; Pizcueta and Luscinskas, 1994; Tedder et al., 1995; Stadtmann et al., 2013; Zuchtriegel et al., 2015). We found considerable L-selectin levels on all circulating myeloid cell subtypes, including neutrophils and monocytes, in uninjured WT mice. After SCI, L-selectin was dynamically regulated on myeloid cells in blood. Notably, there was a 25.3% reduction of L-selectin on neutrophils 24 h after SCI, which may reflect a negative feedback mechanism to attenuate L-selectin-dependent inflammatory activities, as has been described in another setting of injury and inflammation (Strausbaugh et al., 1999). Reduced L-selectin has been previously observed in circulating neutrophils in spinal cord-injured human patients (Bao et al., 2008); however, this study also found reduced L-selectin levels on circulating monocytes. The increase in L-selectin on circulating monocyte populations in our study in mouse may reflect species differences in L-selectin regulation during inflammatory responses to SCI.

L-selectin deficiency was associated with partially reduced neutrophil accumulation into the acutely injured spinal cord (24 h post-SCI). This is consistent with the participation of L-selectin in recruitment of neutrophils, e.g., as an adhesive molecule in secondary tethering to other leukocytes or as a signaling molecule in activating integrins on neutrophils (Walcheck et al., 1996; Zarbock et al., 2011; Stadtmann et al., 2013; Morikis et al., 2017). At 72 h post-SCI, when neutrophil numbers in the cord were ∼3-fold fewer than at 24 h (reflecting death or exit of the infiltrated neutrophils), there was no difference between WT and KO mice. However, at the same time point, we did find reduced oxidative stress in the acutely injured cord of L-selectin KO mice, suggesting the possibility of ROS as a component of L-selectin mediated pathogenesis. Many studies have established a role for L-selectin in modulating the internal signaling and secretome of neutrophils (Zarbock and Ley, 2008), including the potentiation of ROS production (Crockett-Torabi et al., 1995). Elucidation of the signaling pathways by which L-selectin contributes to ROS production after SCI could identify novel targets to reduce acute secondary pathogenesis.

Myelin sheaths of CNS, but not peripheral nervous system, express ectopic ligands (presumed to be carbohydrate-based and structurally related to true biological ligands) for L-selectin that are sufficient to support leukocyte adhesion in an *in vitro* assay (Huang et al., 1991; Huang et al., 1994). We found that neutrophils that infiltrated the injured spinal cord at 24 and 72 h post-SCI retained 19.2% and 14.8% of L-selectin levels, respectively, relative to those in peripheral blood. These levels are still appreciable, since L-selectin is normally present at high density on blood leukocytes (∼10^5^ molecules/cell; Simon et al., 1992). Infiltrating neutrophils and monocytes could potentially interact with these “illegitimate” ligands via L-selectin to facilitate activation of effector functions that promote myelin degradation, a mechanism that has been invoked in a model of experimental allergic encephalitis (Grewal et al., 2001). Notably, engagement of L-selectin on neutrophils with incidental carbohydrate ligands (carcinoma or saliva mucins) potentiates the degranulation of these cells (Shao et al., 2011), even after considerable shedding of L-selectin (Mohanty et al., 2015). Thus, reduced L-selectin on spinal cord-infiltrated neutrophils may still be sufficient to drive post-recruitment activities.

NSAIDs are a heterogeneous group of compounds that continue to be an important intervention in patients with non-severe inflammatory disorders. Several NSAIDs provide neuroprotection in experimental models of SCI (Kwon et al., 2011). In the present study, we show that administration of diclofenac led to marked improvements in long-term recovery and sparing of white matter after SCI. The beneficial effects of diclofenac treatment following SCI were comparable to those seen in L-selectin KO mice. Our study thus adds diclofenac, a widely prescribed medication (Altman et al., 2015), to the group of NSAIDs that are beneficial in SCI.

We were initially drawn to diclofenac because it belongs to the subgroup of NSAIDs that are capable of inducing a high level of L-selectin shedding from the surface of leukocytes (Díaz-González et al., 1995; Gómez-Gaviro et al., 2002). In the present study, diclofenac induced the loss of L-selectin in non-classical monocytes and neutrophils within the blood and spinal cord of injured mice. Our report is the first to show a differential effect of NSAIDs on loss of L-selectin from monocyte subtypes with non-classical monocytes being the susceptible population in the context of SCI. The diclofenac-induced reduction in L-selectin on non-classical monocytes could potentially impact signaling by these cells and diminish their deleterious activities in SCI (Donnelly et al., 2011). With respect to neutrophils, the diclofenac-induced reduction of L-selectin levels was not accompanied by reduced accumulation in the injured spinal cord (24 h), which contrasts with the observations in L-selectin KO mice at this time point. This difference may be ascribed to the fact that diclofenac treatment resulted in only a partial loss of L-selectin. Nonetheless, the diclofenac treatment reduced oxidative stress at 72 h post-SCI, an effect that could plausibly be a consequence of reduced signaling and degranulation of neutrophils, as detailed above.

The anti-inflammatory activities of NSAIDs are usually attributed to inhibition of cyclooxygenase (COX), a key enzyme for prostaglandin production. However, Sánchez-Madrid, Díaz-González, and co-workers have also highlighted L-selectin shedding from neutrophils as a potential anti-inflammatory action of certain NSAIDs (Herrera-García et al., 2013; Díaz-González and Sánchez-Madrid, 2015). In our experiments, we cannot exclude that the benefit of diclofenac is due to COX inhibition or diclofenac-induced shedding of molecules other than L-selectin. However, our finding of equivalent neurologic outcomes after SCI in L-selectin KO mice, treated with diclofenac compared to the vehicle control group, suggests that the beneficial effect of diclofenac is related to its ability to induce L-selectin shedding. Taken together, our diclofenac findings are consistent with the possibility that the partial loss of L-selectin induced by this drug reduces the deleterious activities of myeloid cells in secondary pathogenesis. Further mechanistic studies are needed to substantiate this scenario as opposed to other potential activities of diclofenac.

In our experiments, 40 mg/kg of diclofenac was required to improve long-term neurologic recovery after SCI, but repeated dosing at this high level was associated with increased mortality. According to the FDA’s guidelines for conversion to a human equivalent dose (Nair and Jacob, 2016), the dose for a 60-kg individual would be 3.25 mg/kg. In fact, oral administration of diclofenac to human subjects at a dose of ∼2.5 mg/kg/d has been shown to promote robust loss of L-selectin on blood neutrophils (Baranda, 1998). Thus, it would be feasible to test diclofenac for efficacy in human SCI at an FDA-approved dose. Evaluation of diclofenac in the context of sex as a biological variable will be an important factor in such studies.

Our findings establish a therapeutic window for diclofenac administration with efficacy observed up to at least 3 h, but <8 h, after SCI. This timeframe is consistent with our observations that the pathologic contributions of L-selectin are associated with early myeloid cell activities (<72 h post-SCI). Recent clinical studies have also highlighted the importance of early intervention with anti-inflammatory strategies (Ahuja et al., 2016). Future exploration of more clinically relevant methods for delivery of diclofenac and development of faster acting structural analogues with improved safety profiles may extend the therapeutic window for strategies targeting L-selectin after SCI.

## Synthesis

Reviewing Editor: Jun Chen, University of Pittsburgh Decisions are customarily a result of the Reviewing Editor and the peer reviewers coming together and discussing their recommendations until a consensus is reached. When revisions are invited, a fact-based synthesis statement explaining their decision and outlining what is needed to prepare a revision will be listed below. The following reviewer(s) agreed to reveal their identity: Nick Jeffery, Angela Boutte.

Both reviewers acknowledged the potential significance of the findings presented in the current manuscript. However, they indicated that appropriate revisions are required to clarify some of the missing experimental details, and, also, presentation of concepts, rationale, and results should be improved. Their specific points are copied below.

Reviewer #1

General comments

Generally, this is a well-written and clearly described series of experiments to address the question as to whether L-selectin on neutrophils may be a suitable target in acute spinal cord injury. The data generally support this notion, although there are some details of study design that lack complete clarity. Specific items here include the need to: i) explain how animal numbers for each experiment were derived and how much drop-out there was because of death etc; ii) explain the gating strategy in flow cytometry. Some of the illustrations are not of good quality (in the review copy at least).

Specific comments (by line numbers where relevant):

138: transverse sections presumably?

266: what were the power analyses? It helps a little to have some further explanation - mainly regarding what type of magnitude of difference you were looking for in various experiments. It would be helpful to know how large a difference in various outcomes you considered important to detect.

Fig 1d is poorly lit - can this be improved?

289-291: this wasn't described as an outcome measure in the M&M and so it would be appropriate to highlight that this was an exploratory analysis.

Fig 3: the values shown in B and C don't seem to tally well - or am I misreading this?

Fig 4a - need to change scale on the y-axis so readers can see the small values better

Fig 5: need to keep axes the same scale for comparable figures eg E and C & D

Fig 6: there is a strong time effect here that needs comment. There is no change in CD62L between 2 and 24 hours in the diclofenac group - it's just that there is a change in the vehicle group that suggests an effect of diclofenac. I think this needs some discussion in the discussion section.

Fig 7 - need to change axis scale on a c and e

Fig 7 b & 407-408: as mentioned above - this is not a test specified in M&M

404-407: The Basso mouse scale is not really a continuous scale. While the analysis assuming that this is a continuous measure is widely accepted it might also be helpful for at least one of the experiments to show how many mice change category (on BMS) - and by how many categories - between different time points because this is what is really driving the (pseudo) mean difference. Perhaps you could include a table for these data here showing the range of scores at 1, 7 and 35 days for each group?

Fig 7F - overexposed - can this be improved?

Comparison of Fig 10 with Fig 7 - and within Fig 10 - suggests a difference in scores in vehicle animals that is quite large (almost as large as the score change for diclofenac versus control). I realize how this can happen but it probably deserves some comment in the discussion.

Reviewer #2

This work has the capability to advance our knowledge of SCI induced secondary injury responses that are controlled, in part, by cells of myeloid lineage. Further, the authors provide evidence of a potentially powerful mechanism (L-selectin sheddding) impacted by a well-known NSAID, diclofenac.

However, the overall theme and main purpose of the study as well as key outcomes are a bit lost, since the paper is not well structured to present the data in a clear manner. The manuscript introduction, methods and discussion should be more concise and there are several grammatical/stylistic errors.

For instance, the abstract and the introduction, and discussion do not provide specific rationale for testing diclofenac. Stating FDA approval is insufficient and a clear mechanism must be cited or suggested based on the data shown. The inclusion of MDA (as opposed to other markers) and the analysis of blood as it relates specifically to SCI is is not specific or clear, and one cannot assume direct relationships between blood derived immune cells vs those that invade the SCI lesion without empirical evidence (e.g. GFP bone marrow transplants). This point is also relevant to resident immune cells in the spinal cord that express the same markers and may shed l-selectin. Abundant overarching references to general nsaids and other adhesion should be limited .

Overall, the manuscript would benefit from a clear, focused theme in the text. The figures present a novel biomarker and mechanism of secondary SCI responses and present a feasible therapeutic strategy. The role of peripheral immune cells may be downplayed (or a separate manuscript) in favor of focusing on the spinal cord, which is of immediate interest to neuroscience audiences.
